# Occlusal traits of 4–5‐year‐old Estonians. Parents' perception of orthodontic treatment need and satisfaction with dental appearance

**DOI:** 10.1002/cre2.170

**Published:** 2019-01-31

**Authors:** Hettel Sepp, Mare Saag, Heli Vinkka‐Puhakka, Anna‐Liisa Svedström‐Oristo, Timo Peltomäki

**Affiliations:** ^1^ Department of Stomatology, Faculty of Medicine University of Tartu Tartu Estonia; ^2^ Department of Oral Development and Orthodontics, Institute of Dentistry University of Turku Turku Finland; ^3^ Oral and Maxillofacial Unit, Tampere University Hospital, and Faculty of Medicine and Life Sciences, University of Tampere, and Institute of Dentistry, Faculty of Health Sciences University of Eastern Finland Kuopio Finland

**Keywords:** occlusal traits, perception, satisfaction, treatment need

## Abstract

This study aims to evaluate the prevalence of occlusal traits and to assess parents'/caregivers' satisfaction with child's dental appearance and perception of orthodontic treatment need in 4–5‐year‐old Estonians. Clinical records and plaster casts of 390 children (190 girls and 200 boys, mean age 4.7 years, range 4 – 5 years) were analyzed. Assessed occlusal traits included deciduous canine and second molar sagittal relationship, overjet, overbite, crowding, midline diastema, crossbite, and scissor bite. Parents'/caregivers' opinions regarding their child's teeth were determined with a questionnaire. The most prevalent occlusal traits were symmetrical sagittal relationship in deciduous canines (78.2%) and molars (75.1%), Class I sagittal relationship in deciduous canines (69.7%) and midline diastema (67.7%). Asymmetrical sagittal canine relationship was registered in 21.8% deciduous canines and in 24.9% second deciduous molars. Parents'/caregivers' perceived orthodontic treatment need was related to Class III sagittal relationship in canines, increased overjet and overbite, negative overbite, and crossbite. Prevalence of most occlusal traits in Estonian children were in line with those reported in neighboring countries. Parents/caregivers were well able to observe occlusal traits that deviated from acceptable occlusion.

## 1 INTRODUCTION

Childhood is an important period in growth and development of the craniofacial area and teeth. Fully erupted deciduous dentition provides prognostic features from the standpoint of the future development of permanent dentition. The benefit of guiding interceptive interventions and preventive measures in deciduous and mixed dentition has been debated for several decades (Bishara, Hoppens, Jakobsen, & Kohout, 1988; Freeman, 1977; Hixon, 1968; Lavelle, 1976; Leighton, 1970; Sonnesen, Bakke, & Solow, 2001; Thilander, Rubio, Pena, & de Mayorga, 2002). Marked individual variation in growth and development of the jaw, however, complicates the prognosis of occlusal development (Amini, Hamedi, Haji Ghadimi, & Rakhshan, 2017; Horowitz & Hixon, 1966; Leighton, 1975; Solow, 1980; Thilander, 2009).

Moorrees (1959) has provided a baseline analysis of longitudinal dental development between ages 3 and 18. Cross‐sectional studies of occlusal traits in different age groups give an overall picture of dental development in the population and assist in recognizing the individuals in need of closer follow‐up (Brunelle, Bhat, & Lipton, 1996; Thilander, Pena, Infante, Parada, & de Mayorga, 2001).

Nevertheless, it has recently been shown that, in addition to secular trends that influence dental development, there are also population‐specific occlusal traits (Eskeli et al., 2016; Kerosuo, Laine, Nyyssonen, & Honkala, 1991).

This study is the third in a series of cross‐sectional investigations analyzing the prevalence of occlusal traits in Estonians between the ages of 4 and 21 years.

The aims of this study were to evaluate

- The distribution of occlusal traits in Estonian 4–5‐year‐olds.
- Parents'/caregivers' satisfaction with their children's dental appearance and their perceptions on orthodontic treatment need in this age group.

Work hypotheses of this study were that

- The prevalence and types of occlusal traits in Estonia do not differ significantly from those in neighboring countries in the age group of 4–5 years.
- Occlusal traits observed by parents/caregivers differ from those observed by orthodontic professionals.

## 2 SUBJECTS AND METHODS

### 2.1 Data source

A 95% confidence interval around an estimate (±2.5% of the estimate) was specified for sample size calculation. In the sampling, a multistage stratified cluster design was implemented. Recruitment of 4–5‐year‐old children was started in March 2011 and completed in January 2012. All of the 4–5‐year‐old children from 11 selected kindergartens—five in North Estonia, four in Central Estonia, and two in Southwest Estonia—were invited to participate in the study.

**Figure 1:**
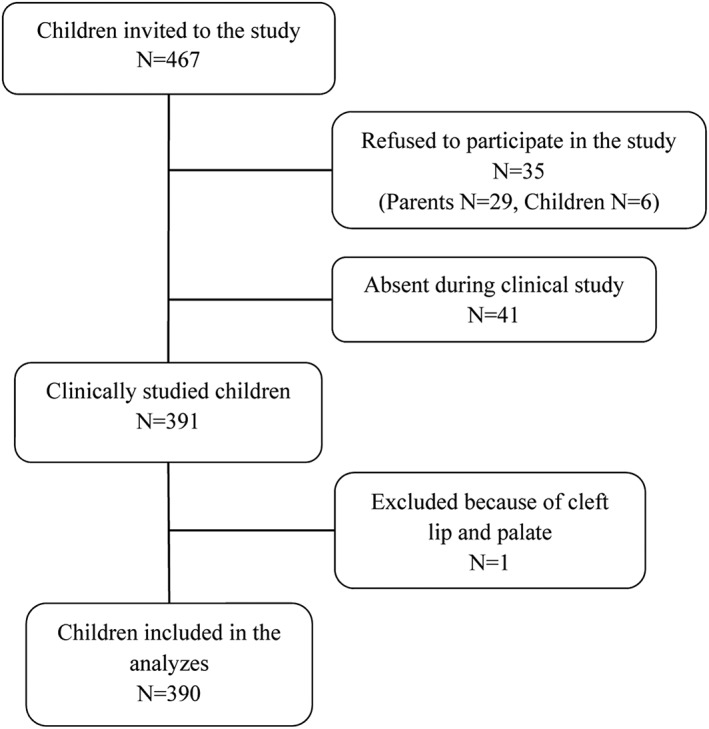
The flow chart describing refining of the final sample

The number of invited children was 467. A total of 77 children were excluded for following reasons: (a) 41 children were not in kindergarten on the examination days, (b) 29 parents did not agree their child to participate in the clinical study (c) six children were too afraid to participate in the clinical study, and (d) one child had cleft lip and palate. Thus, the final sample consists of 390 children (190 girls and 200 boys, mean age 4.7 years, range 4–5 years). The sampling procedure is illustrated in Figure 1.

Prior to the study, a written description of the study protocol was given to all parents/caregivers. All parents/caregivers signed an informed consent form. The study protocol was approved by the Ethics Review Committee on Human Research of the University of Tartu (Protocol No. 186T‐24).

### 2.2 Registration of occlusal traits

The following occlusal traits were registered clinically in centric occlusion by one orthodontist (Examiner 1): (a) sagittal relationships in deciduous canines and second molars separately for right and left side, (b) overjet (OJ), (c) overbite (OB), (d) crossbite, and (e) scissor bite.

To obtain centric occlusion, a child was asked to open mouth only slightly. The orthodontist gently verified that mandible was relaxed, then the child was asked to bite together.

The examination was carried out in the kindergarten's medical office using a dental mirror, probe, pencil (0.3 mm), and millimeter ruler (with 0.5 mm intervals; Dentaurum 042‐751 Münchner Modell). The clinical study was complemented with alginate impressions for plaster casts. Preshaped bite registration wax was softened in warm water bath and placed against the upper dental arch; relaxed mandible was gently guided into centric occlusion to get indentations of cusps of lower teeth into registration wax.

Examiners 1 and 2 registered three features from the plaster casts in consensus: (a) end‐to‐end relationship of the deciduous canines and second molars, separately for the right and left side, (b) crowding, and (c) diastemas between central incisors.

Registration of the occlusal traits was based on international standards (Brunelle et al., 1996; Horowitz & Hixon, 1966; Moorrees, 1959). A detailed description of the criteria has been presented previously (Sepp, Saag, Svedström‐Oristo, Peltomäki, & Vinkka‐Puhakka, 2017).

### 2.3 Questionnaire

Opinions regarding children's general dental health, tooth alignment, dental appearance, and orthodontic treatment need were collected with a questionnaire filled in at home by parents/caregivers. More than one answer per question was allowed. The questionnaire was modified from one used in a previous study (Pietilä & Pietilä, 1996).

### 2.4 Reliability and statistical analysis

Twenty‐two children were reexamined clinically by Examiner 1 after a 1‐week interval before the intended study. The reliability was very good (*r* > 0.99). A total of 122 plaster casts were reexamined for calibration after 1 month by Examiners 1 and 2 together. The reliability was very good (*r* > 0.98). Chi^2^ and Fisher's exact test (where necessary) were used to compare the frequencies of occlusal traits (IBM SPSS v.20 software for Windows [IBM Corp, Armonk, NY, USA]). *p* values of less than 0.05 were considered statistically significant. The test–retest was calculated using Pearson's correlations (*r* = 0.72, *p* < 0.01).

## 3 RESULTS

In the current study, there were a total of 28 children (7.2%) with symmetrical flush terminal plane and Class I in deciduous canines, OJ 1–3 mm and OB 1–3 mm, no crowding, scissor bite or crossbite. Of their parents/caregivers, 23 (85.2%) were satisfied with the alignment of teeth.

### 3.1 Occlusal traits

The most prevalent occlusal traits were symmetrical relationship in deciduous canines (78.2%) and molars (75.1%), Class I sagittal relationship in deciduous canines (69.7%) and midline diastema (67.7%) (Table 1).

**Table 1:** Prevalence of occlusal traits in 4–5‐year‐old Estonian children (*N* = 390)

Occlusal trait	Prevalence (%; *N* = 390)
Deciduous molar relationship	
Mesial terminal plane	47.9
Flush terminal plane	42.8
Distal terminal plane	33.6
Symmetric	75.1
Asymmetric	24.9
Canine relationship	
Class I	69.7
End‐to‐end	42.8
Class II	5.6
Class III	3.8
Symmetric	78.2
Asymmetric	21.8
Horizontal relationship	
Overjet ≥ 3.5 mm	15.6
Overjet < 0 mm	2.3
Vertical relationship	
Overbite ≥ 3.5 mm	38.7
Overbite < 0 mm	3.1
Transversal relationship	
Posterior crossbite	17.4
Scissor bite	0.5
Midline diastema	
Upper and lower arch	34.9
Maxillary	46.9
Mandibular	55.6
Crowding	
Upper and lower arch	0.0
Maxillary	0.0
Mandibular	0.3

**Figure 2:**
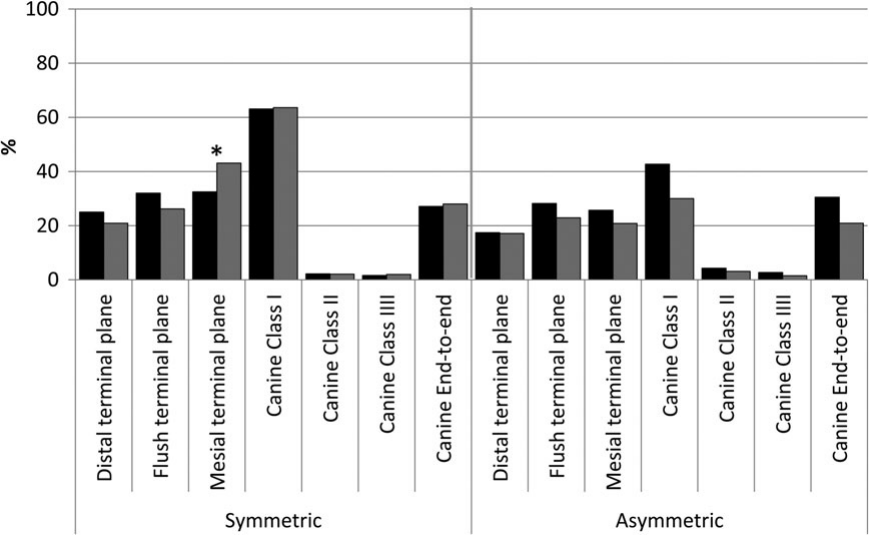
Distribution of sagittal relationship of the second deciduous molars and deciduous canines in 4–5‐year‐old Estonian girls (black) and boys (gray) (*N* = 390). Gender difference was present only for mesial terminal plane (*p < 0.05)

Asymmetrical sagittal relationship in deciduous canines was found in 21.8% and in second deciduous molars in 24.9% of the examinees (Figure 2). Children with asymmetric end‐to‐end sagittal relationship in canines had statistically significantly more crossbites compared with children with symmetric end‐to‐end sagittal relationship (*p* < 0.01).

**Figure 3:**
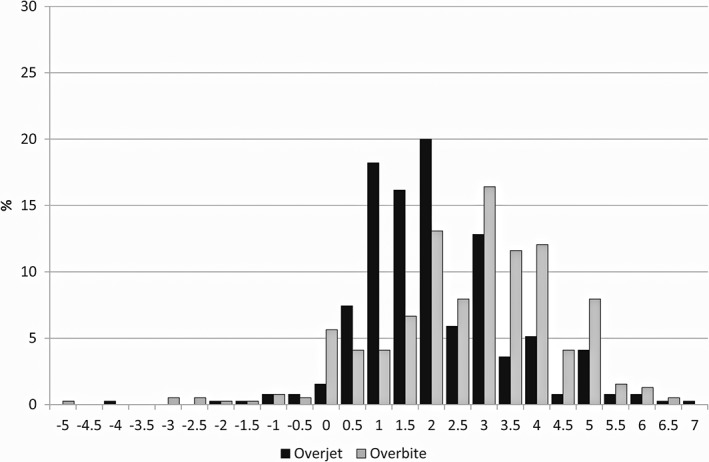
The range of overjet (black) and overbite (gray) in 4–5‐year‐old children in Estonia (*N* = 390)

The OJ ranged from −4.0 mm to 7.0 mm (mean 2.1 mm, *SD* 1.4) and the OB from −5.0 mm to 6.5 mm (mean 2.7 mm, *SD* 1.7) (Figure 3). A statistically significant gender difference was found in OJ. Boys had on average larger OJ than girls (boys 2.2 ± 1.4 vs. girls 1.9 ± 1.4, *p* < 0.01), and there was a trend of more boys with increased OJ (OJ ≥ 3.5 mm) compared with girls (*p* = 0.06).

Of children with negative OJ (OJ < 0 mm), 24.2% had Class III sagittal relationship in canines unilaterally or bilaterally. OJ and OB were statistically significantly larger in children with distal terminal plane (OJ 1.8 vs. 2.6, OB 2.4 vs. 3.3, *p* < 0.01) compared with those with Class II sagittal relationship in canines (OJ 2.0 vs. 3.1, OB 2.6 vs. 4.0, *p* < 0.01), end‐to‐end canine sagittal relationship (OJ 1.8 vs. 2.5, OB 2.5 vs. 3.0, *p* < 0.01), and those without crossbite (OJ 2.2 vs. 1.2, OB 3.1 vs. 1.2, *p* < 0.01).

The midline diastema ranged from 0.1 to 6.0 mm. In the lower arch, it was statistically significantly smaller in children with distal terminal plane of deciduous molars (0.5 vs. 0.7, *p* = 0.02), Class III sagittal relationship in canines (0.6 vs. 1.1, *p* = 0.01), and those with crossbite (0.6 vs. 0.8, *p* = 0.03).

Posterior crossbite was observed in 6.7% on the right side, in 4.3% on the left side, and in 6.4% on both sides. One child had a scissor bite on the right and one on the left side. None of the children had bilateral scissor bite.

### 3.2 Parents'/caregivers' satisfaction

The children whose parents/caregivers were satisfied with the arrangement of their child's teeth had significantly less scissor bite (*p* = 0.02), increased OB (*p* = 0.01), negative OB (*p* < 0.01), and Class III sagittal relationship in canines (*p* = 0.05), compared with children whose parents were dissatisfied with the arrangement of their child's teeth (Table 2).

**Table 2:** Prevalence of occlusal traits and parents'/caregivers' opinions regarding their children's dental health and the appearance and alignment of their teeth (*N* = 390)

	Girl		Boy		Total	
	*N*	%	*N*	%	*N*	%
Prevalence of occlusal traits						
Posterior crossbite	31	16.3	37	18.5	68	17.4
Overjet <0 mm	6	3.2	3	1.5	9	2.3
Overbite <0 mm	5	2.6	7	3.5	12	3.1
Overjet ≥4 mm	18	9.5	29	14.5	47	12.1
Overbite ≥4 mm	54	28.4	53	26.5	107	27.4
Canine Class III	8	4.2	7	3.5	15	3.8
Satisfaction with child's dental health						
Very satisfied	44	23.2	25	12.5	69	17.7
Satisfied	112	58.9	120	60.0	232	59.5
I do not care	1	0.5	2	1.0	3	0.8
Dissatisfied	26	13.7	40	20.0	66	16.9
Not satisfied at all	5	2.6	11	5.5	16	4.1
I do not know	2	1.1	2	1.0	4	1.0
Total	190	100.0	200	100.0	390	100.0
Satisfaction with the alignment and appearance of child's teeth						
Very satisfied	40	21.1	29	14.5	69	17.7
Satisfied	114	60.0	143	71.5	257	65.9
Dissatisfied	21	11.1	17	8.5	38	9.7
Unhappy	0	0.0	1	0.5	1	0.3
I do not know	14	7.4	6	3.0	20	5.1
No answer	1	0.5	4	2.0	5	1.3
Total	190	100.0	200	100.0	390	100.0
Desire for orthodontic treatment						
Definitely not	18	9.5	14	7.0	32	8.2
No, I do not think so	119	62.6	125	62.5	244	62.6
Yes, I think so	36	18.9	33	16.5	69	17.7
Yes, definitely	1	0.5	3	1.5	4	1.0
No answer	16	8.4	25	12.5	41	10.5
Total	190	100.0	200	100.0	390	100.0

### 3.3 Parents'/caregivers' opinions on orthodontic treatment need in 4–5‐year‐old children

All parents whose child had Class III sagittal relationship in canines, increased OJ (OJ threshold ≥4 mm) and OB (OB threshold ≥4 mm), negative OB, and crossbite thought their child was in need of orthodontic treatment.

Reduction in the amount of caries was highlighted most often by parents/caregivers as a reason for orthodontic treatment (52.5%). They were also more likely to want improvement in function if the children had crossbite (27.0% vs. 16.0%, *p* = 0.01) (Table 3).

**Table 3:** The reasons for parents' desire for orthodontic treatment in 4–5‐year‐old Estonian children (*N* = 390)

Reason	*N*	%
To reduce the amount of caries	84	52.5
To improve dental appearance	27	16.9
To improve occlusal function	21	13.1
Other reason	17	10.6
To facilitate cleaning	11	6.9
Total	160	100.0

## 4 DISCUSSION

Data on distribution of occlusal traits, on parents'/caregivers' satisfaction with dental appearance, and their opinions on orthodontic treatment need in 4–5‐year‐old children have been lacking in Estonia.

In Estonia, the prevalence of mesial terminal plane was much higher than in Finland (47.9% vs. 19.1%, respectively) (Keski‐Nisula, Lehto, Lusa, Keski‐Nisula, & Varrela, 2003). Variability in deciduous molar sagittal relationship may be partly related to subjectivity in its definition. Indeed, the canine sagittal relationship has proved to be more reliable than that of molars. Although the prevalence of Class III sagittal relationship in canines was lower in Estonia than in Sweden (3.8% vs. 9.0%) (Dimberg, Lennartsson, Söderfeldt, & Bondemark, 2013), it was higher than in Finland (1.5%) (Keski‐Nisula et al., 2003). However, these values may possibly include some canine Class I relationship because all the occlusal traits in our study were assessed using central occlusion as the reference. In fact, at the age of 4–5 years, ongoing development of temporomandibular joints makes definition of centric relation more or less unreliable (Karlo et al., 2010).

Distribution of symmetric and asymmetric sagittal molar relationships in Estonian children was in line with that of Finnish children (Keski‐Nisula et al., 2003).

In our study, the prevalence of posterior crossbite was significantly higher in children with asymmetrical than symmetrical sagittal relationship. Prevalence of bilateral crossbite in Estonians was equal to that of Swedes (Dimberg et al., 2013) but higher than that of Finnish children (Keski‐Nisula et al., 2003). However, the prevalence of negative OJ in this study was similar to that in Finland as well as Sweden (Dimberg et al., 2013; Keski‐Nisula et al., 2003).

The prevalence of midline diastema in 4–5‐year‐olds (67.7%) reflected that of 7–10‐year‐old Estonians (73.0%) (Sepp et al., 2017). This finding conforms with the idea that the structure of the frenulum influences the position of central incisors.

Estonian children had a lower prevalence of increased OJ (OJ > 4 mm) compared with Finnish and Swedish children (Dimberg et al., 2013; Keski‐Nisula et al., 2003). Instead, prevalence of increased OB (OB > 4 mm) (27.0%) of Estonian children was in line with that of Finnish children (34.0%) (Keski‐Nisula et al., 2003).

There was no crowding in any of the studied 4–5‐year‐old Estonians. This finding is contrary to the situation in Finland, where crowding in the maxilla has been found in 11.6% and in the mandible in 38.9% of children (Keski‐Nisula et al., 2003). The difference was clear, although crowding was measured on plaster casts in both of these studies.

Benefit of orthodontic treatment is estimated by a dentist using professional criteria. Patients/parents/caregivers make their own observations, which are equally important in judgment of treatment need and outcome. Therefore, it is important to know how parents/caregivers, as laypersons, observe dentition and how critical they are (Ryan & Cunningham, 2018).

### What this paper adds?

- Prevalence of most occlusal traits in Estonian 4–5‐year‐olds is in line with those reported in neighboring countries, except for negative OB, increased OJ, and lack of crowding.
- The most prevalent occlusal traits in Estonian 4–5‐year‐olds are symmetrical sagittal relationship in deciduous canines and molars, Class I sagittal relationship in deciduous canines, mesial terminal plane in deciduous second molars, and midline diastema.
- With regard to dental health and appearance, more than four out of five parents/caregivers are satisfied. Dissatisfied parents seem to focus on occlusal traits like negative OB, deep bite, and Class III relationship in canines.
- The hypothesis that parents/caregivers do not pay attention to professionally important traits in their child's dentition is rejected.

### Why this paper is important for dentists?

- It is important to know that the majority of 4–5‐year‐old children have occlusal traits that may develop into malocclusion.
- Dental professionals should appreciate parents'/caregivers' observations regarding their child's occlusal traits and functioning of the masticatory system. They seem to be well able to observe occlusal traits and functions that deviate from the so‐called “normal.”
